# Does the sex of one’s co-twin affect height and BMI in adulthood? A study of dizygotic adult twins from 31 cohorts

**DOI:** 10.1186/s13293-017-0134-x

**Published:** 2017-04-27

**Authors:** Leonie H. Bogl, Aline Jelenkovic, Eero Vuoksimaa, Linda Ahrenfeldt, Kirsi H. Pietiläinen, Maria A. Stazi, Corrado Fagnani, Cristina D’Ippolito, Yoon-Mi Hur, Hoe-Uk Jeong, Judy L. Silberg, Lindon J. Eaves, Hermine H. Maes, Gombojav Bayasgalan, Danshiitsoodol Narandalai, Tessa L. Cutler, Christian Kandler, Kerry L. Jang, Kaare Christensen, Axel Skytthe, Kirsten O. Kyvik, Wendy Cozen, Amie E. Hwang, Thomas M. Mack, Catherine A. Derom, Robert F. Vlietinck, Tracy L. Nelson, Keith E. Whitfield, Robin P. Corley, Brooke M. Huibregtse, Tom A. McAdams, Thalia C. Eley, Alice M. Gregory, Robert F. Krueger, Matt McGue, Shandell Pahlen, Gonneke Willemsen, Meike Bartels, Toos C. E. M. van Beijsterveldt, Zengchang Pang, Qihua Tan, Dongfeng Zhang, Nicholas G. Martin, Sarah E. Medland, Grant W. Montgomery, Jacob v. B. Hjelmborg, Esther Rebato, Gary E. Swan, Ruth Krasnow, Andreas Busjahn, Paul Lichtenstein, Sevgi Y. Öncel, Fazil Aliev, Laura A. Baker, Catherine Tuvblad, Sisira H. Siribaddana, Matthew Hotopf, Athula Sumathipala, Fruhling Rijsdijk, Patrik K. E. Magnusson, Nancy L. Pedersen, Anna K. Dahl Aslan, Juan R. Ordoñana, Juan F. Sánchez-Romera, Lucia Colodro-Conde, Glen E. Duncan, Dedra Buchwald, Adam D. Tarnoki, David L. Tarnoki, Yoshie Yokoyama, John L. Hopper, Ruth J. F. Loos, Dorret I. Boomsma, Thorkild I. A. Sørensen, Karri Silventoinen, Jaakko Kaprio

**Affiliations:** 1 Institute for Molecular Medicine FIMM, University of Helsinki, P.O. Box 20, FI-00014 Helsinki, Finland; 2 Department of Public Health, University of Helsinki, Helsinki, Finland; 3 Department of Social Research, University of Helsinki, Helsinki, Finland; 4 Department of Genetics, Physical Anthropology and Animal Physiology, University of the Basque Country UPV/EHU, Leioa, Spain; 5 Department of Public Health, Epidemiology, Biostatistics & Biodemography, The Danish Twin Registry, University of Southern Denmark, Odense, Denmark; 6 Obesity Research Unit, Research Programs Unit, University of Helsinki, Helsinki, Finland; 7 Endocrinology, Abdominal Center, Helsinki University Central Hospital and University of Helsinki, Helsinki, Finland; 8 Istituto Superiore di Sanità–National Center for Epidemiology, Surveillance and Health Promotion, Rome, Italy; 9 Department of Education, Mokpo National University, Jeonnam, South Korea; 10 Department of Human and Molecular Genetics, Virginia Institute for Psychiatric and Behavioral Genetics, Virginia Commonwealth University, Richmond, VA USA; 11 Department of Human and Molecular Genetics, Psychiatry & Massey Cancer Center, Virginia Commonwealth University, Richmond, VA USA; 12 Healthy Twin Association of Mongolia, Ulaanbaatar, Mongolia; 13 Graduate School of Biomedical and Health Sciences, Hiroshima University, Hiroshima, Japan; 14 The Australian Twin Registry, Centre for Epidemiology and Biostatistics, The University of Melbourne, Melbourne, VIC Australia; 15 Department of Psychology, Medical School Berlin, Berlin, Germany; 16 Department of Psychiatry, University of British Columbia, Vancouver, BC Canada; 17 Department of Clinical Biochemistry and Pharmacology and Department of Clinical Genetics, Odense University Hospital, Odense, Denmark; 18 Department of Clinical Research, University of Southern Denmark, Odense, Denmark; 19 Odense Patient data Explorative Network (OPEN), Odense University Hospital, Odense, Denmark; 20 Department of Preventive Medicine, Keck School of Medicine of USC, University of Southern California, Los Angeles, CA USA; 21 USC Norris Comprehensive Cancer Center, Los Angeles, CA USA; 22 Centre of Human Genetics, University Hospitals Leuven, Leuven, Belgium; 23 Department of Obstetrics and Gynaecology, Ghent University Hospitals, Ghent, Belgium; 24 Department of Health and Exercise Sciences and Colorado School of Public Health, Colorado State University, Fort Collins, USA; 25 Psychology and Neuroscience, Duke University, Durham, NC USA; 26 Institute for Behavioral Genetics, University of Colorado, Boulder, CO USA; 27 Institute of Psychiatry, Psychology & Neuroscience, MRC Social, Genetic & Developmental Psychiatry Centre, King’s College London, London, UK; 28 Department of Psychology, Goldsmiths, University of London, London, UK; 29 Department of Psychology, University of Minnesota, Minneapolis, MN USA; 30 Department of Biological Psychology, VU University Amsterdam, Amsterdam, Netherlands; 31 Department of Noncommunicable Diseases Prevention, Qingdao Centers for Disease Control and Prevention, Qingdao, China; 32 Institute of Public Health, Epidemiology, Biostatistics and Biodemography, University of Southern Denmark, Odense, Denmark; 33 Department of Public Health, Qingdao University Medical College, Qingdao, China; 34 Genetic Epidemiology Department, QIMR Berghofer Medical Research Institute, Brisbane, Australia; 35 Molecular Epidemiology Department, QIMR Berghofer Medical Research Institute, Brisbane, Australia; 36 Department of Medicine, Stanford Prevention Research Center, Stanford University School of Medicine, Stanford, CA USA; 37 Center for Health Sciences, SRI International, Menlo Park, CA USA; 38 HealthTwiSt GmbH, Berlin, Germany; 39 Department of Medical Epidemiology and Biostatistics, Karolinska Institutet, Stockholm, Sweden; 40 Department of Statistics, Faculty of Arts and Sciences, Kırıkkale University, Kırıkkale, Turkey; 41 Psychology and African American Studies, Virginia Commonwealth University, Richmond, USA; 42 Faculty of Business, Karabuk University, Karabuk, Turkey; 43 Department of Psychology, University of Southern California, Los Angeles, CA USA; 44 School of Law, Psychology and Social Work, Örebro University, Örebro, Sweden; 45 Institute of Research & Development, Battaramulla, Sri Lanka; 46 Faculty of Medicine & Allied Sciences, Rajarata University of Sri Lanka, Saliyapura, Sri Lanka; 47 Institute of Psychiatry Psychology and Neuroscience, NIHR Mental Health Biomedical Research Centre, South London and Maudsley NHS Foundation Trust and King’s College London, London, UK; 48 Research Institute for Primary Care and Health Sciences, School for Primary Care Research (SPCR), Faculty of Health, Keele University, Staffordshire, UK; 49 Institute of Gerontology and Aging Research Network–Jönköping (ARN-J), School of Health and Welfare, Jönköping University, Jönköping, Sweden; 50 Department of Human Anatomy and Psychobiology, University of Murcia, Murcia, Spain; 51 IMIB-Arrixaca, Murcia, Spain; 52 Department of Developmental and Educational Psychology, University of Murcia, Murcia, Spain; 53 QIMR Berghofer Medical Research Institute, Brisbane, Australia; 54 Washington State Twin Registry, Washington State University–Health Sciences Spokane, Spokane, WA USA; 55 Department of Radiology, Semmelweis University, Budapest, Hungary; 56 Hungarian Twin Registry, Budapest, Hungary; 57 Department of Public Health Nursing, Osaka City University, Osaka, Japan; 58 Department of Epidemiology, School of Public Health, Seoul National University, Seoul, South Korea; 59 The Charles Bronfman Institute for Personalized Medicine, The Mindich Child Health and Development Institute, Icahn School of Medicine at Mount Sinai, New York, NY USA; 60 Novo Nordisk Foundation Centre for Basic Metabolic Research (Section on Metabolic Genetics), and Department of Public Health, Faculty of Health and Medical Sciences, University of Copenhagen, Copenhagen, Denmark; 61 Institute of Preventive Medicine, Bispebjerg and Frederiksberg Hospitals, Copenhagen, The Capital Region Denmark; 62 Osaka University Graduate School of Medicine, Osaka University, Osaka, Japan

**Keywords:** Prenatal hormone exposure, Opposite-sex twins, Height, Body mass index, CODATwins

## Abstract

**Background:**

The comparison of traits in twins from opposite-sex (OS) and same-sex (SS) dizygotic twin pairs is considered a proxy measure of prenatal hormone exposure. To examine possible prenatal hormonal influences on anthropometric traits, we compared mean height, body mass index (BMI), and the prevalence of being overweight or obese between men and women from OS and SS dizygotic twin pairs.

**Methods:**

The data were derived from the COllaborative project of Development of Anthropometrical measures in Twins (CODATwins) database, and included 68,494 SS and 53,808 OS dizygotic twin individuals above the age of 20 years from 31 twin cohorts representing 19 countries. Zygosity was determined by questionnaires or DNA genotyping depending on the study. Multiple regression and logistic regression models adjusted for cohort, age, and birth year with the twin type as a predictor were carried out to compare height and BMI in twins from OS pairs with those from SS pairs and to calculate the adjusted odds ratios and 95% confidence intervals for being overweight or obese.

**Results:**

OS females were, on average, 0.31 cm (95% confidence interval (CI) 0.20, 0.41) taller than SS females. OS males were also, on average, taller than SS males, but this difference was only 0.14 cm (95% CI 0.02, 0.27). Mean BMI and the prevalence of overweight or obesity did not differ between males and females from SS and OS twin pairs. The statistically significant differences between OS and SS twins for height were small and appeared to reflect our large sample size rather than meaningful differences of public health relevance.

**Conclusions:**

We found no evidence to support the hypothesis that prenatal hormonal exposure or postnatal socialization (i.e., having grown up with a twin of the opposite sex) has a major impact on height and BMI in adulthood.

**Electronic supplementary material:**

The online version of this article (doi:10.1186/s13293-017-0134-x) contains supplementary material, which is available to authorized users.

## Background

Studies in litter-bearing mammals have found that hormones can transfer from one fetus to adjacent fetuses in the uterus [1]. Fetuses located between two males have increased concentrations of testosterone compared to fetuses located between two females [2], and such fetuses are heavier, suggesting that the intrauterine position may influence metabolic set points involved in the regulation of body weight and fat storage [3].

Whether an analogous effect exists in humans is uncertain; however, it has been postulated that twins influence each other hormonally during prenatal life because they share their intrauterine environment [4]. This is called the twin testosterone transfer (TTT) hypothesis. In particular, females who develop with a male co-twin are potentially exposed to higher levels of prenatal testosterone, the most potent androgen, than females from same-sex (SS) twin pairs [5]. Support for the TTT hypothesis comes from previous twin studies showing that females from opposite-sex (OS) twin pairs express more male-typical characteristics (i.e., are masculinized) in a variety of sexually dimorphic traits. One phenotype with a replicated finding of masculinization of females with a male co-twin is the Mental Rotation Test, which measures a specific cognitive ability with a large sex difference favoring males [6, 7]. However, also postnatal socialization effects need to be considered as an alternative explanation to the TTT hypothesis. Having grown up with a sibling of the same sex versus a sibling of the opposite sex is likely to result in different social learning experiences and has been linked to sex-typed behavior; for example, boys with older brothers and girls with older sisters have been found to be more sex typed than children from opposite-sex sibling dyads [8].

Among the few studies that have specifically compared anthropometric and health-related variables, some differences between females from OS and SS twin pairs have been reported, for example, for height [9], birth weight [10], body mass index (BMI) [11], dyslipidemia [11], and leukocyte telomere lengths [12]. However, it is important to note that null reports also exist for several traits. For example, there are no differences between OS and SS female twins with regard to the prevalence of polycystic ovary syndrome [13] or hormone-related cancers [14]. Similarly, males from SS twin pairs could be masculinized compared with OS males due to additional testosterone exposure of the male co-twin, although most studies that have investigated co-twin effects in males have failed to identify differences between OS and SS male twins [5, 15].

Many previous studies are constrained by several limiting factors, including small sample size, lack of replication, and the inclusion of monozygotic (MZ) twins in the SS groups, which is questionable due to the variation in placentation patterns between MZ and dizygotic (DZ) twin pairs [5]. Furthermore, publication bias is likely to have occurred, with non-significant results being less likely to be published than significant ones [16]. In 2011, Tapp et al. reviewed the evidence that fetuses gestated with a male co-twin are masculinized during the development due to prenatal androgen exposure and concluded that while accumulated evidence lacks consistency, it is sufficient to warrant further research, ideally using large samples of OS and SS twin pairs [15].

To this end, the present study aims to test whether two sexually dimorphic anthropometric traits, height and BMI and the prevalence of overweight and obesity differ between females and males from OS and SS DZ twin pairs. Consistent with the TTT hypothesis, OS females are predicted to be taller and have a higher BMI than SS females, and SS males are predicted to be taller and have a higher BMI than OS males. The sample was drawn from the newly established CODATwins (Collaborative project of Development of Anthropometrical measures in Twins) database [17], which, being based on original data, avoids publication bias and is very large and able to address the hypothesis with much greater power than previous studies.

## Methods

### Sample

The CODATwins project is a major international twin collaboration that was initiated in 2013 to pool data on zygosity, weight, and height from twin cohorts across the world. A description of the project and the participating twin cohorts was reported previously in detail [17]. The main CODATwins database includes 960,859 height and weight measures from both MZ and DZ twins. Extreme outliers and biologically implausible values for height and BMI were inspected visually for each age decade and by sex, resulting in elimination of less than 0.2% of the observations from the original database. For the present study, we excluded (1) all measurements prior to the age of 20 years to avoid confounding by pubertal stage; (2) MZ twins (*n* = 165,305); (3) DZ twin individuals with missing information on OS or SS status; (4) cohorts which did not collect data from OS twins; and (5) cohorts for which only a few OS twins were available, i.e., if the ratio of OS to SS DZ twins was <0.15 (the expectation being unity as sex is determined independently in the two fetuses). As a sensitivity analysis, excluding eight cohorts with 2866 twins with a ratio below 0.6 rather than 0.15 did not change the results (data available on request). Furthermore, if there were multiple observations for an individual, we restricted the analyses to one observation per individual by using the observation at the youngest age. The effect sizes were virtually unchanged when using older observations as opposed to younger observations, and therefore, only the latter results are presented. In total, 31 cohorts from 19 countries met our inclusion criteria (Additional file 1: Tables S1 and S2). The final sample for the present analysis consisted of 122,302 DZ twin individuals of which 68,494 were from SS and 53,808 from OS twin pairs. The median age (and interquartile range) of the participants was 44.0 (28.7–56) years for males and 42.0 (28.0–55.9) years for females (Additional file 1: Table S1 for females and Additional file 1: Table S2 for males show these descriptive statistics by twin type and cohort). In order to examine age effects, the sample was divided into younger (<50 years) and older (50+ years) age groups, which is a proxy for menopausal status in women. Among the 66,956 females and 55,346 males, 63 and 62%, respectively, were classified as younger adults. Height and weight were almost all self-reported (97%). BMI calculated as body weight in kilograms divided by height in meters squared (kg/m^2^) was used as an indicator of adiposity. Zygosity was determined by questionnaires or DNA genotyping depending on the study [17].

### Statistical analysis

The statistical analyses were performed using Stata (version 13.0, Stata Corporation, TX, USA) where *p* < 0.05 was considered statistically significant. Descriptive statistics for the OS and SS twins are reported as means and standard deviations (SD) separately for females and males. Multiple regression analysis with the twin type (i.e., opposite versus same sex) as a predictor was carried out to compare height and BMI in twins from OS pairs with those from SS pairs. Regression coefficients are shown with their 95% confidence intervals (CIs). Marginal means, i.e., adjusted for cohort, age, and birth year, are presented by twin type along with CIs. Logistic regression models adjusted for cohort, age, and birth year were used to calculate the adjusted odds ratios (OR) and 95% CIs for being overweight or obese with the twin type as a predictor. WHO Asian BMI cut-off points (overweight ≥23 kg/m^2^ and obese ≥27.5 kg/m^2^) were used for Asian cohorts, and WHO standard BMI cut-off points (overweight ≥25 kg/m^2^ and obese ≥30 kg/m^2^) were applied for all other cohorts (from Europe, North-America, and Australia) [18]. In all regression models, SS twins were set as the reference group. The non-independence (clustering) within twin pairs was taken into account in both multiple and logistic regression analyses by using the “cluster” option in Stata to yield robust estimators of variance [19].

To assess heterogeneity across cohorts, a random-effects meta-analysis with inverse variance weighting derived from the DerSimonian and Laird estimator was performed using the user-written “metan” command in Stata and visualization of forest plots. The *I* ^2^ statistic was used to examine variability in effect sizes between cohorts. The *I* ^2^ statistic estimates the proportion of variation in effect sizes due to heterogeneity, whereby values of 25–49, 50–74, and >75% indicate low, moderate, and high heterogeneity, respectively [20].

## Results

Males were taller (mean ± SD 178.42 ± 7.24 versus 164.80 ± 6.78 cm, *p* < 0.001) and had a higher BMI (mean ± SD 25.23 ± 3.63 versus 23.97 ± 4.50 kg/m^2^, *p* < 0.001) than females. Descriptive statistics for age, height, and BMI are shown for the OS and SS female and male twins in Table 1. Both men and women were on average tallest in the Netherlands and shortest in Sri Lanka. Mean BMI was lowest for women from South Korea and men from Japan and highest for African American men and women (Additional file 1: Tables S1 and S2).

**Table 1:** Age and unadjusted height (cm) and BMI (kg/m^2^) in females and males from same- and opposite-sex twin pairs

	Females		Males	
	SS	OS	SS	OS
Number of individuals	39,856	27,100	28,638	26,708
Age				
Mean	43.9	43.0	44.5	43.3
SD	16.9	15.7	16.6	15.6
Min	20.0	20.0	20.0	20.0
Max	99.0	95.9	99.2	94.5
Height				
Mean	164.6	165.0	178.3	178.5
SD	6.8	6.7	7.2	7.2
Min	135.0	135.7	147.0	145.0
Max	193.0	194.0	208.3	207.0
BMI				
Mean	24.0	24.0	25.2	25.3
SD	4.6	4.4	3.6	3.7
Min	13.1	13.3	14.2	13.4
Max	49.9	49.8	47.8	49.8

*SS* same sex, *OS* opposite sex, *SD* standard deviation, *Min* minimum, *Max* maximum, *BMI* body mass index

Female twins with a male co-twin were on average 0.31 cm taller than females with a female co-twin (*p* < 0.0001). Male twins with a female co-twin were 0.14 cm taller than male twins with a male co-twin (*p* = 0.025) (Table 2). There were no significant differences in BMI between OS and SS females or between OS and SS males (Table 2). The estimates did not differ between the younger (*β* = −0.04 kg/m^2^, 95% CI −0.13, 0.05 for females and *β* = −0.01 kg/m^2^, 95% CI −0.09, 0.07 for males) or older groups (*β* = −0.05 kg/m^2^, 95% CI −0.07, 0.16 for females and *β* = −0.06 kg/m^2^, 95% CI −0.03, 0.16 for males) separately.

**Table 2:** Adjusted means and regression coefficient for height (cm) and BMI (kg/m^2^) in females and males from same- and opposite-sex twin pairs

Phenotype	Twin type	Mean	95% confidence intervals	Regression coefficient	95% confidence intervals
Height	SS females	164.7	164.6, 164.8		
	OS females	165.0	164.9, 165.1	0.31 cm	0.20, 0.41
	SS males	178.4	178.3, 178.4		
	OS males	178.5	178.4, 178.6	0.14 cm	0.02, 0.27
BMI	SS females	23.95	23.90, 24.00		
	OS females	24.00	23.05, 24.05	0.05 kg/m^2^	−0.02, 0.12
	SS males	25.20	25.16, 25.25		
	OS males	25.26	25.22, 25.31	0.06 kg/m^2^	0.00, 0.12

The results are adjusted for cohort, age and birth year, and the non-independence (clustering) of observations within twin pairs

*SS* same sex, *OS* opposite sex, *BMI* body mass index

The ORs for overweight and obesity did not differ between OS and SS females nor between OS and SS males (Table 3). When analysed by age group, the ORs for overweight did not differ from 1.00 for the younger (OR = 0.99, 95% CI 0.94, 1.03 for females and OR = 0.99, 95% CI 0.95, 1.04 for males) or older group (OR = 1.05, 95% CI 0.99, 1.11 for females and OR = 1.02, 95% CI 0.96, 1.08 for males). Females from OS twin pairs were at a reduced risk of obesity (OR = 0.90, 95% CI 0.83, 0.97) in the younger but not in the older age group (OR = 0.98, 95% CI 0.90, 1.07), but the 95% CIs for these two estimates were overlapping. For males, there was no difference in obesity risk in either age group (OR = 1.04, 95% CI 0.96, 1.13 in the younger and OR = 1.04, 95% CI 0.95, 1.14 in the older group).

**Table 3:** Prevalence and adjusted odds ratios for overweight and obesity in females and males from same- and opposite-sex twin pairs

Phenotype	Twin type	% overweight/obese	95% confidence intervals	Odds ratio	95% confidence intervals
Overweight	SS females	30.2	29.7, 30.7	1.00 (reference)	
	OS females	30.9	30.3, 31.4	1.03	0.99, 1.07
	SS males	47.0	46.4, 47.7	1.00 (reference)	
	OS males	47.6	47.0, 48.2	1.02	0.99, 1.06
Obesity	SS females	8.45	8.15, 8.76	1.00 (reference)	
	OS females	8.11	7.78, 8.44	0.96	0.90, 1.01
	SS males	8.04	7.70, 8.34	1.00 (reference)	
	OS males	8.48	8.13, 8.83	1.06	1.00, 1.13

The results are adjusted for cohort, age and birth year, and the non-independence (clustering) of observations within twin pairs. Overweight was defined as ≥23 kg/m^2^ and obesity as ≥27.5 kg/m^2^ for Asian cohorts and as BMI ≥25 kg/m^2^ and BMI ≥30 kg/m^2^, respectively, for all other cohorts

*SS* same sex, *OS* opposite sex

**Fig. 1:**
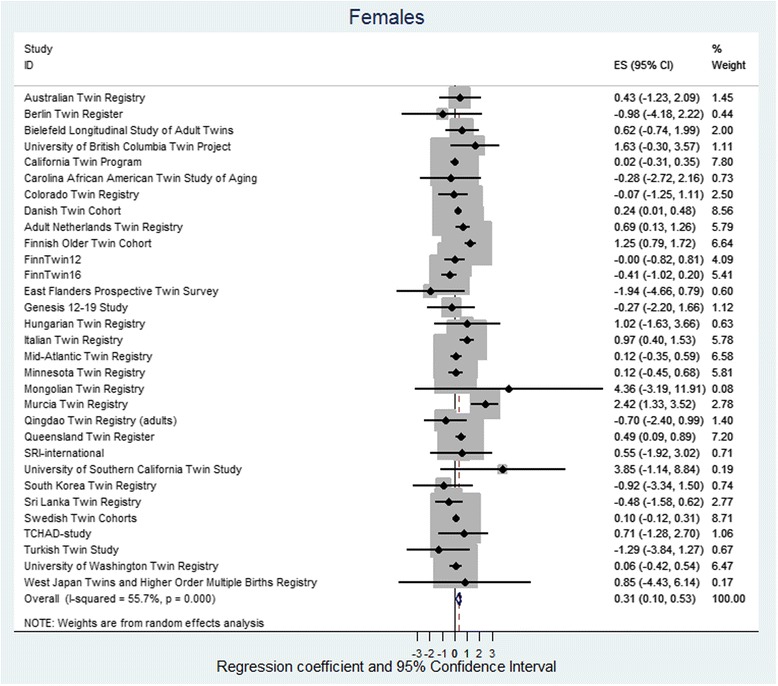
Regression coefficient and 95% CIs using a random-effects model with height as the dependent variable and twin type as the independent variable for females. The effect size shows the increase in height of OS females as compared to dizygotic SS females. If the twin testosterone hypothesis were supported, the effect would be in the positive direction, and the effect size would be significant. The results are adjusted for age and birth year and the non-independence (clustering) of observations within twin pairs. *Squares* indicate study-specific regression coefficients, and the size of the *squares* is proportional to the weight of each study, i.e., the inverse of the variance. The *horizontal lines* represent 95% CIs

**Fig. 2:**
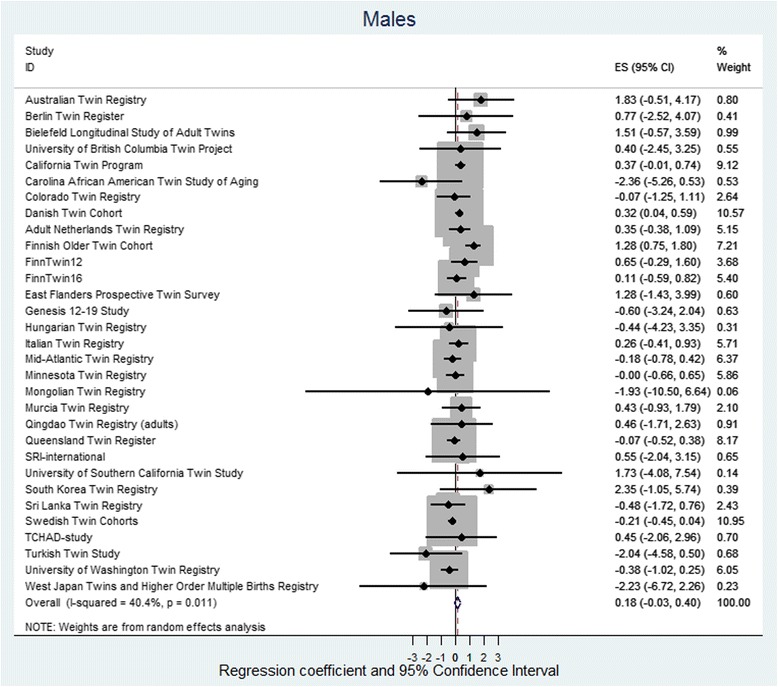
Regression coefficient and 95% CIs using a random-effects model with height as the dependent variable and twin type as the independent variable for males. The effect size shows the increase in height of OS males as compared to dizygotic SS males. If the twin testosterone hypothesis were supported, the effect would be in the negative direction, and the effect size would be significant. The results are adjusted for age and birth year and the non-independence (clustering) of observations within twin pairs. *Squares* indicate study-specific regression coefficients, and the size of the *squares* is proportional to the weight of each study, i.e., the inverse of the variance. The *horizontal lines* represent 95% CIs

**Fig. 3:**
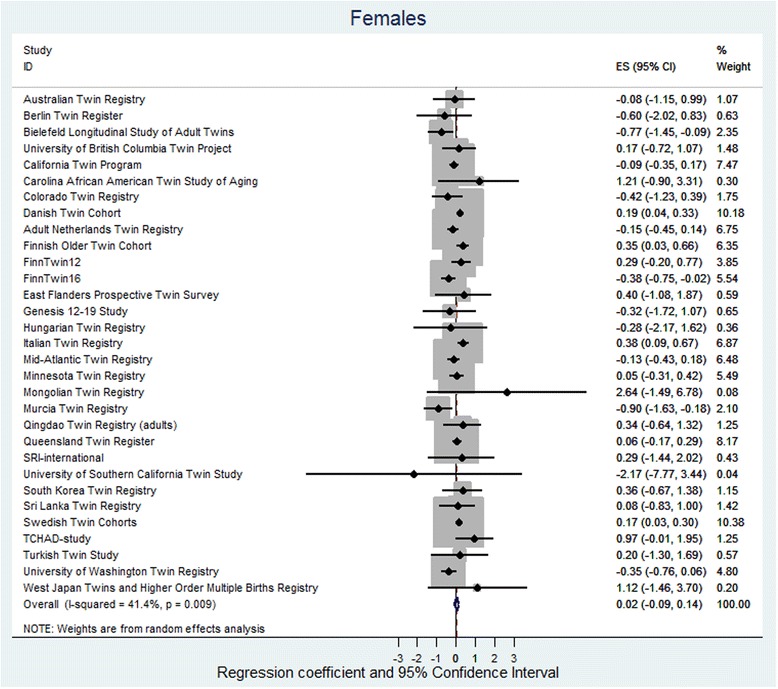
Regression coefficient and 95% CIs using a random-effects model with BMI as the dependent variable and twin type as the independent variable for females. The effect size shows the increase in BMI of OS females as compared to dizygotic SS females. If the twin testosterone hypothesis were supported, the effect would be in the positive direction and the effect size would be significant. The results are adjusted for age and birth year and the non-independence (clustering) of observations within twin pairs. *Squares* indicate study-specific regression coefficients, and the size of the *squares* is proportional to the weight of each study, i.e., the inverse of the variance. The horizontal lines represent 95% CIs

**Fig. 4:**
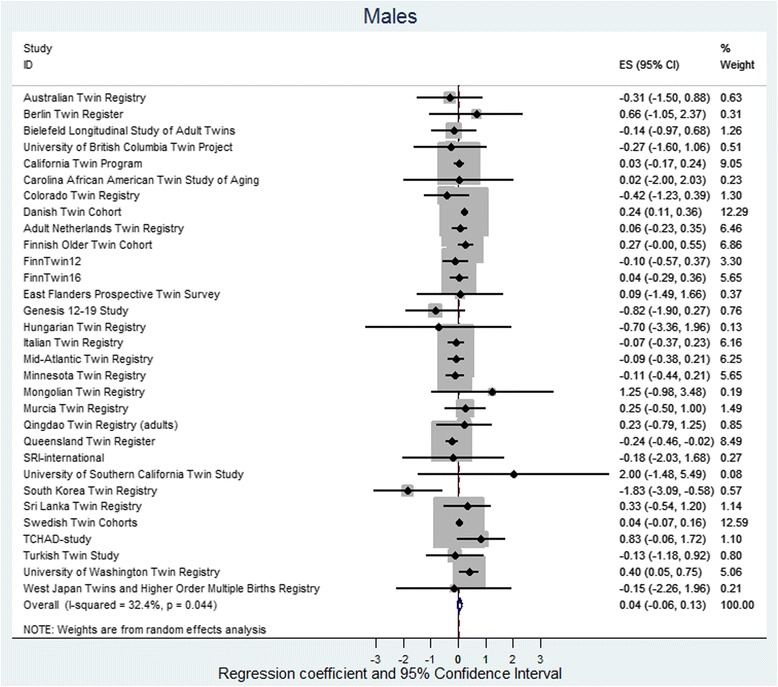
Regression coefficient and 95% CIs using a random-effects model with BMI as the dependent variable and twin type as the independent variable for males. The effect size shows the increase in BMI of OS males as compared to dizygotic SS males. If the twin testosterone hypothesis were supported, the effect would be in the negative direction and the effect size would be significant. The results are adjusted for age and birth year and the non-independence (clustering) of observations within twin pairs. *Squares* indicate study-specific regression coefficients, and the size of the *squares* is proportional to the weight of each study, i.e., the inverse of the variance. The *horizontal lines* represent 95% CIs

The random effect meta-analysis found low to moderate heterogeneity between cohorts, with *I* ^2^ ranging from 32 to 56%. The pooled regression coefficients for height were *β* = 0.31 cm (95% CI 0.10, 0.53) and *β* = 0.18 cm (95% CI −0.03, 0.40) in females and males, respectively (Figs. 1 and 2). For BMI, the corresponding values were *β* = 0.02 kg/m^2^ (95% CI −0.09, 0.14) and *β* = 0.04 kg/m^2^ (95% CI −0.06, 0.13) for females and males, respectively (Figs. 3 and 4).

The height differences between OS and SS twins were not associated with the mean heights of the cohorts, showing that height differences between OS and SS twins were not greater in taller cohorts (Additional file 2: Figure S1). The BMI differences between OS and SS twins were not associated with the mean BMIs of the cohorts for males, but there was an inverse association for females, showing that OS twins tended to have a lower BMI than SS twins with an increasing mean BMI of the cohorts (Additional file 2: Figure S2). The Spearman correlations were not significant with the exception of BMI for females. Overall, no consistent pattern was observed.

## Discussion

Using this newly established large-scale international twin collaboration, we tested the TTT hypothesis and its possible long-lasting influence on adult height and BMI and find little evidence in support of the hypothesis.

There were no differences in mean BMI or the prevalence of overweight or obesity between twins with a co-twin of the opposite sex and twins with a co-twin of the same sex. Consistent with the TTT hypothesis, we observed that females with a male co-twin were taller than females with a female co-twin. However, we also observed that males with a female co-twin were slightly taller than males with a male co-twin, a finding that is opposite in direction to that predicted by the TTT hypothesis. Yet, our finding for males is in accordance with previous observations on birth weight in twins. Males from OS twin pairs have higher growth rates starting from week 32 of gestation [21] and a higher mean birth weight when compared with males from SS pairs [21, 22]. The increased birth weight of males with a female co-twin as compared to males with a male co-twin has been ascribed to the longer duration of gestation of male-female pairs [22] and the more successful in utero competition for nutrients of males in the presence of a female, rather than a male, co-twin [23]. Birth weight is an established determinant of adult height [24], and thus, differences in fetal growth and duration of gestation that depend on the co-twin’s sex could also contribute to the small height differences observed in the present analyses.

Furthermore, explanations other than the in utero environment need to be considered. First, postnatal socialization effects could also contribute to differences in anthropometric traits between OS and SS twins, since growing up with a sibling of the same or opposite sex is likely to be different through sibling and parental interactions. However, in previous studies of non-twin siblings, height in childhood was unrelated to the sex of the sibling [25]. Second, because DZ twins are slightly taller and heavier than MZ twins [26], misclassification of a small fraction of MZ twins as SS DZ twins could have contributed to part of the observed small differences between twin-type groups. The misclassification rate of questionnaire-based zygosity falls below 5% when compared with genetic markers [27].

Support for the theory that prenatal exposure to hormones has long-lasting effects on physiology and behavior in later life comes mainly from animal models, in which prenatal androgen exposure has been examined by injecting the developing fetuses with varying doses of testosterone [28–32]. These animal studies have provided evidence that prenatal androgen exposure during the organizational period induces long-term alterations in metabolic function. Alteration of the epigenome during fetal development might be an underlying mechanism linking prenatal androgenization and endocrine disorders in adulthood, such as the polycystic ovary syndrome (PCOS) [33]. Prenatal androgenization produces features of the metabolic syndrome in rodents, including increased body weight and visceral adiposity, impaired insulin secretion, and hepatic triglyceride content [29, 30]. These alterations could be partly mediated by food intake, as androgenization of female rats in the neonatal period leads to a feeding pattern in adulthood that is more similar to that of male rats, including diminished meal number and increased meal size and food intake as well as increased weight [34]. Female rhesus monkeys exposed to increased levels of testosterone during early-to-mid gestation develop metabolic abnormalities found in PCOS women, including insulin resistance, increased visceral fat accumulation, impaired pancreatic beta-cell function, and type 2 diabetes [28, 31, 35]. Interestingly, the effect of prenatal androgen excess is not only restricted to females but is also present in prenatally androgenized male monkeys, who displayed by insulin resistance and impaired pancreatic beta-cell function despite no changes in adult androgen levels [32].

Extrapolating findings from experimental animal studies to humans is complicated by a variety of factors that differ across species, including the markedly different placentation patterns and duration of pregnancy [1]. Measuring prenatal androgen exposure in humans is inherently difficult. Amniocentesis, in which a small amount of fluid is sampled from the amniotic sac surrounding the developing fetus, provides the most informative measurement of prenatal androgen exposure. It is, however, invasive and carries a risk of miscarriage and should therefore only be performed when there is medical need [15]. The comparison of twins from OS and SS pairs is considered a proxy measure of prenatal hormone exposure, as hormone transfer in twin pregnancies could occur through one of the following routes: the maternal-fetal route (through the maternal bloodstream) or the fetal-fetal route (diffusion across fetal membranes). A relatively small study did not detect differences in maternal serum steroid levels between mothers who were expecting OS or SS DZ twins, making the maternal route less plausible [5]. At birth, females with a twin brother are not exposed to higher androgen concentrations compared with females with a twin sister [36]. Other studies of singletons have found that maternal serum testosterone concentrations differ between women carrying male and female fetuses [37, 38] and that maternal and fetal testosterone are positively correlated [39]. Thus, it is likely that human sex steroids are transferred between twins, but direct evidence for the hormone transfer to occur in humans does not exist.

Multiple twin studies have studied whether fetuses gestated with a male co-twin are masculinized in development, and results on perception, cognition, physiological, and morphological outcomes are more consistent with the TTT hypothesis than those on behavioral traits, including, for example, disordered eating, sensation seeking and aggression [15]. Relatively few twin studies have studied whether anthropometry and health outcomes differ between OS and SS twins. Among recent studies, Ahrenfeldt et al. [14] found no significant differences in hormone-dependent cancer incidence between OS and SS twins using a large sample of Nordic twins. Alexanderson et al. [11] found no differences in height, while weight, BMI, and the prevalence of dyslipidemia were higher for women from OS pairs than SS pairs older than age 60 years using the Screening Across the Lifespan Twin study, which is also included in the CODATwins project as part of the Swedish twin studies. Loehlin and Martin [9] reported a marginally significant increased height for females from OS compared with females from SS pairs. Gaist et al. [40] found no differences in any of the strength and anthropometric measures for middle-aged female twins from the Danish population-based twin registry.

The main strengths of the present study are that it includes data from 122,302 dizygotic twin individuals from 31 twin studies in 19 countries, increasing the generalizability of the findings and reducing the type 2 error. Furthermore, the narrow confidence intervals indicate high precision. There was low to moderate heterogeneity across cohorts, suggesting relatively consistent findings across studies. Height is a classic example of a sexually dimorphic trait; on average, men are taller than women in all human populations [41–43]; thus, the large sex difference makes it worth the investigation in relation to the prenatal hormone transfer hypothesis. As the sexual dimorphism is greater for body composition and body fat distribution than for BMI [44], we acknowledge the lack of more detailed adiposity measures as a limitation of this study. Zygosity was self-reported and not verified by DNA testing in the majority of studies. That almost all heights and weights were self-reported is another limitation, since perception of body weight has been reported to depend on sibling’s weight and sibling’s weight perceptions, and these relationships have been found to differ by the sex of the sibling [45]. We further acknowledge the lack of data on pubertal timing, a key developmental period that could affect adult weight and height. A recently published study reports the height and BMI differences between OS and SS twins to depend on breastfeeding status. Never-breastfed SS twins tended to be shorter and lighter than never-breastfed OS twins, but breastfed SS twins were consistently taller and heavier than breastfed OS twins throughout adolescence and early adulthood [46]. We recognize the lack of early-life nutritional data as another limitation, and more research is undoubtedly warranted in this field.

## Conclusions

Results from this large international twin collaboration show that females with a twin brother were on average 0.31 cm taller than females with a twin sister. Males with a twin sister were also on average slightly taller than males with a twin brother, but this height difference was only 0.14 cm. Although statistically significant, the observed differences were of small magnitudes and therefore of limited public health importance. Mean BMI and the prevalence of being overweight or obese did not differ between males and females from SS and OS twin pairs. Thus, the present findings provide no evidence to support the hypothesis that in utero exposure to testosterone or postnatal socialization (i.e., having grown up with a twin of the opposite sex) has a major impact on height and BMI in later life. However, these results do not rule out the possibility that OS and SS twins differ in height or BMI at other developmental periods such as prenatal development, early infancy, or during puberty.

## Additional files

Additional file 1: Table S1. Sample size, mean, standard deviation and range for age by cohort in females from same- and opposite-sex dizygotic twin pairs. **Table S2.** Sample size, mean, standard deviation, and range for age by cohort in males from same- and opposite-sex dizygotic twin pairs. **Table S3.** Sample size, mean, and standard deviation for height (cm) and BMI (kg/m^2^) by cohort in females from same- and opposite-sex dizygotic twin pairs. **Table S4.** Sample size, mean, and standard deviation for height (cm) and BMI (kg/m^2^) by cohort in males from same- and opposite-sex dizygotic twin pairs. (DOCX 45 kb)

Additional file 2: Figure S1. The differences in height (cm) between opposite sex (OS) and same sex (SS) plotted against the mean heights (cm) of the cohorts for females and males. The figure examines whether the difference in height between OS and SS twins is greater in taller cohorts. The Spearman correlations are *r* = −0.16 and *p* = 0.39 for females and *r* = 0.10 and *p* = 0.59 for males. **Figure S2.** The differences in BMI (kg/m^2^) between opposite-sex (OS) and same-sex (SS) twins plotted against the mean BMIs (kg/m^2^) of the cohorts for females and males. The figure examines whether the difference in BMI between OS and SS twins is greater in heavier cohorts. The Spearman correlations are *r* = −0.39 and *p* = 0.029 in females and *r* = 0.10 and *p* = 0.61 for BMI in males. (DOCX 159 kb)

## Abbreviations

BMI: Body mass index
CI: Confidence interval
CODATwins: COllaborative project of Development of Anthropometrical measures in Twins
DZ: Dizygotic
MZ: Monozygotic
OR: Odds ratio
OS: Opposite sex
SD: Standard deviation
SS: Same sex
TTT: Twin testosterone transfer

## References

[1] Ryan BC, Vandenbergh JG (2002). Intrauterine position effects. Neurosci Biobehav Rev.

[2] vom Saal FS (1989). Sexual differentiation in litter-bearing mammals: influence of sex of adjacent fetuses in utero. J Anim Sci.

[3] Kinsley C, Miele J, Wagner CK, Ghiraldi L, Broida J, Svare B (1986). Prior intrauterine position influences body weight in male and female mice. Horm Behav.

[4] Miller EM (1994). Prenatal sex hormone transfer: a reason to study opposite-sex twins. Personal Individ Differ.

[5] Cohen-Bendahan CC, van de Beek C, Berenbaum SA (2005). Prenatal sex hormone effects on child and adult sex-typed behavior: methods and findings. Neurosci Biobehav Rev.

[6] Vuoksimaa E, Kaprio J, Kremen WS, Hokkanen L, Viken RJ, Tuulio-Henriksson A (2010). Having a male co-twin masculinizes mental rotation performance in females. Psychol Sci.

[7] Heil M, Kavsek M, Rolke B, Beste C, Jansen P (2011). Mental rotation in female fraternal twins: evidence for intra-uterine hormone transfer?. Biol Psychol.

[8] Rust J, Golombok S, Hines M, Johnston K, Golding J, ALSPAC Study Team (2000). The role of brothers and sisters in the gender development of preschool children. J Exp Child Psychol.

[9] Loehlin JC, Martin NG (1998). A comparison of adult female twins from opposite-sex and same-sex pairs on variables related to reproduction. Behav Genet.

[10] Glinianaia SV, Magnus P, Harris JR, Tambs K (1998). Is there a consequence for fetal growth of having an unlike-sexed cohabitant in utero?. Int J Epidemiol.

[11] Alexanderson C, Henningsson S, Lichtenstein P, Holmang A, Eriksson E (2011). Influence of having a male twin on body mass index and risk for dyslipidemia in middle-aged and old women. Int J Obes (Lond).

[12] Benetos A, Dalgard C, Labat C, Kark JD, Verhulst S, Christensen K (2014). Sex difference in leukocyte telomere length is ablated in opposite-sex co-twins. Int J Epidemiol.

[13] Kuijper EA, Vink JM, Lambalk CB, Boomsma DI (2009). Prevalence of polycystic ovary syndrome in women from opposite-sex twin pairs. J Clin Endocrinol Metab.

[14] Ahrenfeldt LJ, Skytthe A, Moller S, Czene K, Adami HO, Mucci LA (2015). Risk of sex-Specific cancers in opposite-sex and same-sex twins in Denmark and Sweden. Cancer Epidemiol Biomarkers Prev.

[15] Tapp AL, Maybery MT, Whitehouse AJ (2011). Evaluating the twin testosterone transfer hypothesis: a review of the empirical evidence. Horm Behav.

[16] Thornton A, Lee P (2000). Publication bias in meta-analysis: its causes and consequences. J Clin Epidemiol.

[17] Silventoinen K, Jelenkovic A, Sund R, Honda C, Aaltonen S, Yokoyama Y (2015). The CODATwins Project: the cohort description of collaborative project of development of anthropometrical measures in twins to study macro-environmental variation in genetic and environmental effects on anthropometric traits. Twin Res Hum Genet.

[18] WHO Expert Consultation (2004). Appropriate body-mass index for Asian populations and its implications for policy and intervention strategies. Lancet.

[19] Williams RL (2000). A note on robust variance estimation for cluster-correlated data. Biometrics.

[20] Higgins JP, Thompson SG, Deeks JJ, Altman DG (2003). Measuring inconsistency in meta-analyses. BMJ.

[21] Melamed N, Yogev Y, Glezerman M (2009). Effect of fetal sex on pregnancy outcome in twin pregnancies. Obstet Gynecol.

[22] Loos RJ, Derom C, Eeckels R, Derom R, Vlietinck R (2001). Length of gestation and birthweight in dizygotic twins. Lancet.

[23] James WH (2002). Gestation and birthweight in dizygotic twins. Lancet.

[24] Allison DB, Paultre F, Heymsfield SB, Pi-Sunyer FX (1995). Is the intra-uterine period really a critical period for the development of adiposity?. Int J Obes Relat Metab Disord.

[25] Lawson DW, Mace R (2008). Sibling configuration and childhood growth in contemporary British families. Int J Epidemiol.

[26] Jelenkovic A, Yokoyama Y, Sund R, Honda C, Bogl LH, Aaltonen S (2015). Zygosity differences in height and body mass index of twins from infancy to old age: a study of the CODATwins Project. Twin Res Hum Genet.

[27] Christiansen L, Frederiksen H, Schousboe K, Skytthe A, von Wurmb-Schwark N, Christensen K (2003). Age- and sex-differences in the validity of questionnaire-based zygosity in twins. Twin Res.

[28] Abbott DH, Tarantal AF, Dumesic DA (2009). Fetal, infant, adolescent and adult phenotypes of polycystic ovary syndrome in prenatally androgenized female rhesus monkeys. Am J Primatol.

[29] Roland AV, Nunemaker CS, Keller SR, Moenter SM (2010). Prenatal androgen exposure programs metabolic dysfunction in female mice. J Endocrinol.

[30] Demissie M, Lazic M, Foecking EM, Aird F, Dunaif A, Levine JE (2008). Transient prenatal androgen exposure produces metabolic syndrome in adult female rats. Am J Physiol Endocrinol Metab.

[31] Abbott DH, Barnett DK, Bruns CM, Dumesic DA (2005). Androgen excess fetal programming of female reproduction: a developmental aetiology for polycystic ovary syndrome?. Hum Reprod Update.

[32] Bruns CM, Baum ST, Colman RJ, Eisner JR, Kemnitz JW, Weindruch R (2004). Insulin resistance and impaired insulin secretion in prenatally androgenized male rhesus monkeys. J Clin Endocrinol Metab.

[33] Xu N, Kwon S, Abbott DH, Geller DH, Dumesic DA, Azziz R (2011). Epigenetic mechanism underlying the development of polycystic ovary syndrome (PCOS)-like phenotypes in prenatally androgenized rhesus monkeys. PLoS One.

[34] Madrid JA, Lopez-Bote C, Martin E (1993). Effect of neonatal androgenization on the circadian rhythm of feeding behavior in rats. Physiol Behav.

[35] Dumesic DA, Schramm RD, Abbott DH (2005). Early origins of polycystic ovary syndrome. Reprod Fertil Dev.

[36] Kuijper EA, Twisk JW, Korsen T, Caanen MR, Kushnir MM, Rockwood AL (2015). Mid-pregnancy, perinatal, and neonatal reproductive endocrinology: a prospective cohort study in twins and singleton control subjects. Fertil Steril.

[37] Meulenberg PM, Hofman JA (1991). Maternal testosterone and fetal sex. J Steroid Biochem Mol Biol.

[38] Harrison RF, Mansfield MD (1980). Maternal plasma androgens in early human pregnancy. Br J Obstet Gynaecol.

[39] Gitau R, Adams D, Fisk NM, Glover V (2005). Fetal plasma testosterone correlates positively with cortisol. Arch Dis Child Fetal Neonatal Ed.

[40] Gaist D, Bathum L, Skytthe A, Jensen TK, McGue M, Vaupel JW (2000). Strength and anthropometric measures in identical and fraternal twins: no evidence of masculinization of females with male co-twins. Epidemiology.

[41] Eveleth PB (1975). Differences between ethnic groups in sex dimorphism of adult height. Ann Hum Biol.

[42] Gray JP, Wolfe LD (1980). Height and sexual dimorphism of stature among human societies. Am J Phys Anthropol.

[43] NCD Risk Factor Collaboration (NCD-RisC). A century of trends in adult human height. Elife. 2016;5. doi:10.7554/eLife.13410. 10.7554/eLife.13410 PMC4961475 27458798

[44] Wells JC (2012). Sexual dimorphism in body composition across human populations: associations with climate and proxies for short- and long-term energy supply. Am J Hum Biol.

[45] Christensen VT (2014). My sibling, my weight. How gender, sibling gender, sibling weight and sibling weight level perception influence weight perception accuracy. Nutr Diabetes.

[46] Kanazawa S, Segal NL (2017). Same-sex twins are taller and heavier than opposite-sex twins (but only if breastfed): possible evidence for sex bias in human breast milk. J Exp Child Psychol.

