# Supplementary material for: Does the sex of one’s co-twin affect height and BMI in adulthood? A study of dizygotic adult twins from 31 cohorts
Source: Biol Sex Differ. 2017 Apr 27;8:14. doi: 10.1186/s13293-017-0134-x (PMC5408365; doi:10.1186/s13293-017-0134-x)
Supplement: Supplementary file 1 — Sample size, mean, standard deviation and range for age by cohort in females from same- and opposite-sex dizygotic twin pairs. Table S2. Sample size, mean, standard deviation, and range for age by cohort in males from same- and opposite-sex dizygotic twin pairs. Table S3. Sample size, mean, and standard deviation for height (cm) and BMI (kg/m2) by cohort in females from same- and opposite-sex dizygotic twin pairs. Table S4. Sample size, mean, and standard deviation for height (cm) and BMI (kg/m2) by cohort in males from same- and opposite-sex dizygotic twin pairs. (DOCX 45 kb) [file 13293_2017_134_MOESM1_ESM.docx]

Additional file 1: Table S1. Sample size, mean, standard deviation and range for age by cohort in females from same- and opposite sex dizygotic twin pairs.

|  | Same-sex females | | | | |  | Opposite-sex females | | | | |
| --- | --- | --- | --- | --- | --- | --- | --- | --- | --- | --- | --- |
| Cohort name | N | Mean | SD | Min | Max |  | N | Mean | SD | Min | Max |
| Australian Twin Registry | 380 | 50.1 | 13.8 | 21.0 | 73.0 |  | 110 | 52.0 | 14.9 | 21.0 | 76.0 |
| Berlin Twin Register | 82 | 35.7 | 12.3 | 20.0 | 68.3 |  | 31 | 32.3 | 8.3 | 20.9 | 56.7 |
| Bielefeld Longitudinal Study of Adult Twins | 358 | 35.3 | 11.2 | 20.0 | 65.0 |  | 93 | 31.5 | 8.8 | 20.0 | 59.9 |
| University of British Columbia Twin Project | 304 | 35.0 | 12.2 | 20.0 | 76.0 |  | 72 | 33.4 | 10.9 | 20.0 | 66.0 |
| California Twin Program | 5520 | 37.5 | 8.7 | 20.0 | 84.0 |  | 3344 | 42.1 | 11.5 | 20.0 | 87.0 |
| Carolina African American Twin Study of Aging | 116 | 48.2 | 13.2 | 25.6 | 78.1 |  | 61 | 44.8 | 13.2 | 22.1 | 84.1 |
| Colorado Twin Registry | 417 | 23.9 | 2.4 | 20.0 | 31.6 |  | 247 | 24.2 | 2.7 | 20.0 | 32.3 |
| Danish Twin Cohort | 7358 | 43.2 | 18.5 | 20.0 | 99.0 |  | 5519 | 39.6 | 14.9 | 20.0 | 91.0 |
| Adult Netherlands Twin Registry | 1640 | 31.4 | 11.6 | 20.0 | 79.0 |  | 987 | 29.0 | 10.4 | 20.0 | 80.0 |
| Finnish Older Twin Cohort | 1216 | 53.2 | 3.7 | 47.1 | 59.9 |  | 1456 | 52.7 | 3.2 | 47.8 | 59.2 |
| FinnTwin12 | 545 | 24.2 | 1.6 | 21.2 | 27.0 |  | 577 | 24.3 | 1.6 | 21.1 | 26.8 |
| FinnTwin16 | 867 | 24.8 | 2.2 | 22.9 | 36.5 |  | 957 | 25.0 | 2.3 | 22.8 | 36.9 |
| East Flanders Prospective Twin Survey | 80 | 26.2 | 3.7 | 20.5 | 32.8 |  | 41 | 28.0 | 4.4 | 20.1 | 31.7 |
| Genesis 12-19 Study | 116 | 24.5 | 1.5 | 20.0 | 27.0 |  | 84 | 24.5 | 1.4 | 20.0 | 28.0 |
| Hungarian Twin Registry | 104 | 46.9 | 15.0 | 20.3 | 74.0 |  | 29 | 41.0 | 15.8 | 20.0 | 71.0 |
| Italian Twin Registry | 1495 | 35.9 | 16.6 | 20.0 | 85.8 |  | 1077 | 35.0 | 16.6 | 20.0 | 85.1 |
| Mid-Atlantic Twin Registry | 2342 | 52.4 | 18.4 | 20.0 | 91.0 |  | 1335 | 48.6 | 17.7 | 20.0 | 93.0 |
| Minnesota Twin Registry | 1749 | 39.9 | 6.6 | 28.0 | 50.1 |  | 916 | 41.9 | 5.0 | 32.8 | 56.5 |
| Mongolian Twin Registry | 8 | 22.7 | 1.5 | 21.0 | 24.9 |  | 10 | 35.8 | 15.3 | 20.2 | 64.7 |
| Murcia Twin Registry | 442 | 52.9 | 7.9 | 41.0 | 69.0 |  | 270 | 57.0 | 6.6 | 45.0 | 73.0 |
| Qingdao Twin Registry (adults) | 98 | 36.9 | 8.9 | 25.0 | 64.0 |  | 94 | 36.8 | 9.1 | 25.0 | 64.0 |
| Queensland Twin Register | 3791 | 34.1 | 12.4 | 20.0 | 90.9 |  | 2213 | 32.7 | 11.9 | 20.0 | 82.6 |
| SRI-international | 122 | 47.0 | 14.3 | 20.0 | 78.8 |  | 42 | 45.8 | 16.0 | 20.0 | 78.5 |
| University of Southern California Twin Study | 17 | 20.6 | 0.6 | 20.1 | 21.9 |  | 19 | 20.7 | 0.6 | 20.0 | 22.0 |
| South Korea Twin Registry | 58 | 22.3 | 1.7 | 20.0 | 26.0 |  | 22 | 21.6 | 1.4 | 20.0 | 24.0 |
| Sri Lanka Twin Registry | 301 | 42.0 | 13.7 | 21.0 | 81.0 |  | 252 | 40.0 | 12.2 | 22.0 | 72.0 |
| Swedish Twin Cohorts | 7973 | 60.2 | 11.2 | 41.5 | 98.0 |  | 5581 | 57.5 | 9.4 | 41.6 | 92.3 |
| TCHAD-study | 76 | 20.3 | 0.2 | 20.0 | 20.5 |  | 57 | 20.3 | 0.1 | 20.0 | 20.5 |
| Turkish Twin Study | 54 | 26.6 | 6.5 | 20.3 | 42.5 |  | 50 | 29.4 | 10.6 | 20.0 | 69.4 |
| Washington State Twin Registry | 2215 | 43.7 | 17.1 | 20.0 | 94.6 |  | 1536 | 43.3 | 17.8 | 20.0 | 95.9 |
| West Japan Twins and Higher Order Multiple Births Registry | 12 | 25.7 | 5.6 | 20.2 | 34.1 |  | 18 | 23.0 | 2.8 | 20.0 | 28.0 |

N, number of twin individuals; SD, standard deviation; Min, minimum; Max, maximum. This descriptive table is not corrected for the non-independence (clustering) of observations within twin pairs.

Additional file 1: Table S2. Sample size, mean, standard deviation and range for age by cohort in males from same- and opposite sex dizygotic twin pairs.

|  | Same-sex males | | | | |  | Opposite-sex males | | | | |
| --- | --- | --- | --- | --- | --- | --- | --- | --- | --- | --- | --- |
| Cohort name | N | Mean | SD | Min | Max |  | N | Mean | SD | Min | Max |
| Australian Twin Registry | 80 | 51.8 | 14.1 | 22.0 | 81.0 |  | 111 | 51.7 | 15.0 | 20.0 | 76.0 |
| Berlin Twin Register | 38 | 36.9 | 12.5 | 21.7 | 63.3 |  | 31 | 32.5 | 8.4 | 20.9 | 56.7 |
| Bielefeld Longitudinal Study of Adult Twins | 94 | 33.6 | 12.2 | 20.0 | 63.0 |  | 93 | 31.5 | 8.8 | 20.0 | 59.0 |
| University of British Columbia Twin Project | 107 | 33.7 | 10.6 | 20.0 | 64.0 |  | 72 | 33.4 | 10.9 | 20.0 | 66.0 |
| California Twin Program | 3734 | 43.7 | 12.1 | 20.0 | 86.0 |  | 3350 | 42.0 | 11.1 | 20.0 | 81.0 |
| Carolina African American Twin Study of Aging | 73 | 48.6 | 11.1 | 25.2 | 76.5 |  | 65 | 45.3 | 12.8 | 22.1 | 84.0 |
| Colorado Twin Registry | 368 | 24.1 | 2.6 | 20.2 | 32.6 |  | 233 | 24.6 | 2.7 | 20.0 | 33.8 |
| Danish Twin Cohort | 6266 | 42.0 | 17.3 | 20.0 | 99.0 |  | 5410 | 40.0 | 15.0 | 20.0 | 91.0 |
| Adult Netherlands Twin Registry | 807 | 29.8 | 12.2 | 20.0 | 81.0 |  | 960 | 29.3 | 10.6 | 20.0 | 80.0 |
| Finnish Older Twin Cohort | 1152 | 53.2 | 3.5 | 47.1 | 59.9 |  | 1456 | 52.8 | 3.2 | 47.8 | 59.2 |
| FinnTwin12 | 456 | 24.1 | 1.6 | 21.2 | 26.7 |  | 480 | 24.2 | 1.6 | 21.1 | 27.5 |
| FinnTwin16 | 826 | 25.2 | 2.7 | 22.9 | 36.8 |  | 885 | 25.2 | 2.7 | 22.9 | 37.4 |
| East Flanders Prospective Twin Survey | 76 | 26.3 | 4.0 | 20.2 | 33.4 |  | 41 | 28.0 | 4.4 | 20.1 | 32.7 |
| Genesis 12-19 Study | 43 | 24.4 | 1.5 | 21.0 | 27.0 |  | 67 | 24.5 | 1.6 | 20.0 | 27.0 |
| Hungarian Twin Registry | 30 | 43.2 | 14.2 | 23.1 | 66.0 |  | 29 | 41.0 | 15.8 | 20.0 | 71.0 |
| Italian Twin Registry | 964 | 35.1 | 17.3 | 20.0 | 83.0 |  | 1074 | 35.0 | 16.6 | 20.0 | 85.2 |
| Mid-Atlantic Twin Registry | 1151 | 46.1 | 17.8 | 20.0 | 92.0 |  | 1334 | 48.6 | 17.7 | 20.0 | 93.0 |
| Minnesota Twin Registry | 1194 | 39.6 | 6.6 | 28.1 | 50.5 |  | 916 | 41.9 | 4.9 | 32.8 | 51.1 |
| Mongolian Twin Registry | 10 | 29.3 | 15.9 | 20.0 | 59.4 |  | 10 | 35.8 | 15.3 | 20.2 | 64.7 |
| Murcia Twin Registry | 322 | 53.3 | 7.2 | 44.0 | 69.0 |  | 270 | 56.9 | 6.7 | 45.0 | 73.0 |
| Qingdao Twin Registry (adults) | 92 | 38.1 | 11.3 | 24.0 | 80.0 |  | 94 | 36.8 | 9.1 | 25.0 | 64.0 |
| Queensland Twin Register | 2154 | 31.7 | 10.9 | 20.0 | 89.7 |  | 2180 | 32.9 | 12.1 | 20.0 | 82.8 |
| SRI-international | 44 | 35.7 | 15.4 | 20.0 | 68.7 |  | 42 | 45.7 | 16.1 | 20.0 | 78.5 |
| University of Southern California Twin Study | 33 | 21.0 | 0.7 | 20.1 | 22.7 |  | 16 | 20.7 | 0.6 | 20.0 | 22.0 |
| South Korea Twin Registry | 28 | 21.2 | 1.9 | 20.0 | 26.0 |  | 22 | 21.6 | 1.4 | 20.0 | 24.0 |
| Sri Lanka Twin Registry | 212 | 39.4 | 12.3 | 22.0 | 73.0 |  | 254 | 40.1 | 12.5 | 22.0 | 77.0 |
| Swedish Twin Cohorts | 6945 | 59.1 | 10.7 | 41.5 | 99.2 |  | 5581 | 57.5 | 9.4 | 41.6 | 92.3 |
| TCHAD-study | 51 | 20.3 | 0.2 | 20.0 | 20.5 |  | 42 | 20.3 | 0.2 | 20.0 | 20.5 |
| Turkish Twin Study | 92 | 32.7 | 10.1 | 20.1 | 53.3 |  | 50 | 29.4 | 10.6 | 20.0 | 69.4 |
| Washington State Twin Registry | 1183 | 44.5 | 17.5 | 20.0 | 89.3 |  | 1524 | 43.4 | 17.8 | 20.0 | 94.5 |
| West Japan Twins and Higher Order Multiple Births Registry | 13 | 22.2 | 1.9 | 20.7 | 26.3 |  | 16 | 22.9 | 2.6 | 20.3 | 28.0 |

N, number of twin individuals; SD, standard deviation; Min, minimum; Max, maximum. This descriptive table is not corrected for the non-independence (clustering) of observations within twin pairs.

Additional file 1: Table S3. Sample size, mean and standard deviation for height (cm) and BMI (kg/m^2^) by cohort in females from same- and opposite sex dizygotic twin pairs.

|  | Same-sex females | | | | |  | Opposite-sex females | | | | |
| --- | --- | --- | --- | --- | --- | --- | --- | --- | --- | --- | --- |
|  |  | Height | | BMI | |  |  | Height | | BMI | |
| Cohort name | N | Mean | SD | Mean | SD |  | N | Mean | SD | Mean | SD |
| Australian Twin Registry | 380 | 164.1 | 7.4 | 25.4 | 5.1 |  | 110 | 164.3 | 8.0 | 25.5 | 5.0 |
| Berlin Twin Register | 82 | 165.4 | 7.8 | 23.9 | 4.1 |  | 31 | 165.0 | 6.8 | 23.0 | 2.9 |
| Bielefeld Longitudinal Study of Adult Twins | 358 | 166.8 | 6.1 | 22.7 | 3.8 |  | 93 | 167.8 | 5.4 | 21.5 | 2.7 |
| University of British Columbia Twin Project | 304 | 164.7 | 7.0 | 22.6 | 4.1 |  | 72 | 166.5 | 7.1 | 22.6 | 3.2 |
| California Twin Program | 5520 | 165.1 | 7.0 | 24.8 | 5.6 |  | 3344 | 164.9 | 6.7 | 25.0 | 5.6 |
| Carolina African American Twin Study of Aging | 116 | 163.3 | 6.9 | 29.0 | 6.8 |  | 61 | 163.3 | 7.8 | 30.3 | 5.7 |
| Colorado Twin Registry | 417 | 165.1 | 6.7 | 23.9 | 5.3 |  | 247 | 165.0 | 7.1 | 23.5 | 4.5 |
| Danish Twin Cohort | 7358 | 166.0 | 6.5 | 23.3 | 4.0 |  | 5519 | 166.7 | 6.3 | 23.3 | 3.9 |
| Adult Netherlands Twin Registry | 1640 | 169.5 | 6.5 | 23.0 | 3.8 |  | 987 | 170.4 | 6.6 | 22.6 | 3.6 |
| Finnish Older Twin Cohort | 1216 | 161.8 | 5.5 | 25.3 | 3.9 |  | 1456 | 163.1 | 5.6 | 25.6 | 4.1 |
| FinnTwin12 | 545 | 165.8 | 6.3 | 22.8 | 3.6 |  | 577 | 165.8 | 6.2 | 23.0 | 4.1 |
| FinnTwin16 | 867 | 166.3 | 6.0 | 22.6 | 3.9 |  | 957 | 165.9 | 5.7 | 22.3 | 3.3 |
| East Flanders Prospective Twin Survey | 80 | 166.6 | 6.0 | 22.0 | 3.3 |  | 41 | 164.9 | 6.8 | 22.5 | 3.8 |
| Genesis 12-19 Study | 116 | 166.2 | 6.9 | 23.5 | 4.8 |  | 84 | 165.9 | 6.1 | 23.1 | 4.3 |
| Hungarian Twin Registry | 104 | 163.7 | 6.8 | 25.5 | 5.5 |  | 29 | 165.6 | 6.2 | 24.0 | 5.1 |
| Italian Twin Registry | 1495 | 162.6 | 6.6 | 21.9 | 3.7 |  | 1077 | 163.7 | 6.9 | 22.2 | 3.8 |
| Mid-Atlantic Twin Registry | 2342 | 163.3 | 6.5 | 23.9 | 4.4 |  | 1335 | 163.6 | 6.4 | 23.7 | 4.3 |
| Minnesota Twin Registry | 1749 | 164.3 | 6.4 | 23.5 | 4.3 |  | 916 | 164.4 | 6.6 | 23.7 | 4.2 |
| Mongolian Twin Registry | 8 | 159.8 | 5.3 | 20.8 | 1.9 |  | 10 | 162.4 | 7.9 | 23.6 | 3.5 |
| Murcia Twin Registry | 442 | 157.7 | 7.0 | 27.0 | 4.8 |  | 270 | 159.2 | 6.1 | 26.9 | 4.4 |
| Qingdao Twin Registry (adults) | 98 | 159.4 | 5.3 | 23.8 | 3.3 |  | 94 | 158.7 | 5.6 | 24.1 | 3.3 |
| Queensland Twin Register | 3791 | 164.0 | 7.2 | 23.1 | 4.1 |  | 2213 | 164.6 | 7.0 | 23.1 | 4.2 |
| SRI-international | 122 | 165.4 | 6.9 | 25.3 | 4.8 |  | 42 | 166.0 | 6.8 | 25.6 | 4.6 |
| University of Southern California Twin Study | 17 | 158.7 | 5.1 | 28.5 | 8.1 |  | 19 | 162.5 | 7.7 | 26.6 | 6.7 |
| South Korea Twin Registry | 58 | 162.5 | 5.0 | 19.5 | 2.0 |  | 22 | 161.1 | 3.8 | 20.0 | 2.0 |
| Sri Lanka Twin Registry | 301 | 152.1 | 6.2 | 24.3 | 5.0 |  | 252 | 152.0 | 6.5 | 24.2 | 4.8 |
| Swedish Twin Cohorts | 7973 | 164.3 | 6.0 | 24.5 | 3.8 |  | 5581 | 164.8 | 6.0 | 24.6 | 3.7 |
| TCHAD-study | 76 | 166.5 | 5.6 | 20.8 | 2.7 |  | 57 | 167.2 | 5.3 | 21.8 | 2.4 |
| Turkish Twin Study | 54 | 164.0 | 5.9 | 21.5 | 3.9 |  | 50 | 162.6 | 6.0 | 22.1 | 2.9 |
| Washington State Twin Registry | 2215 | 164.5 | 6.9 | 26.2 | 6.0 |  | 1536 | 164.6 | 6.8 | 25.9 | 5.8 |
| West Japan Twins and Higher Order Multiple Births Registry | 12 | 156.7 | 6.5 | 19.6 | 2.6 |  | 18 | 156.1 | 5.3 | 20.6 | 1.6 |

BMI, body mass index; N, number of twin individuals; SD, standard deviation.

This descriptive table is not corrected for the non-independence (clustering) of observations within twin pairs.

Additional file 1: Table S4. Sample size, mean and standard deviation for height (cm) and BMI (kg/m^2^) by cohort in males from same- and opposite sex dizygotic twin pairs.

|  | Same-sex males | | | | |  | Opposite-sex males | | | | |
| --- | --- | --- | --- | --- | --- | --- | --- | --- | --- | --- | --- |
|  |  | Height | | BMI | |  |  | Height | | BMI | |
| Cohort name | N | Mean | SD | Mean | SD |  | N | Mean | SD | Mean | SD |
| Australian Twin Registry | 80 | 176.4 | 7.3 | 27.1 | 3.7 |  | 111 | 178.2 | 7.5 | 26.8 | 4.2 |
| Berlin Twin Register | 38 | 176.1 | 5.5 | 25.5 | 3.6 |  | 31 | 177.1 | 6.9 | 25.4 | 3.9 |
| Bielefeld Longitudinal Study of Adult Twins | 94 | 179.2 | 6.4 | 23.7 | 2.9 |  | 93 | 181.0 | 6.7 | 23.4 | 2.8 |
| University of British Columbia Twin Project | 107 | 177.7 | 9.0 | 24.5 | 4.3 |  | 72 | 178.1 | 8.8 | 24.2 | 4.2 |
| California Twin Program | 3734 | 179.4 | 7.2 | 26.4 | 4.0 |  | 3350 | 179.8 | 7.0 | 26.3 | 4.0 |
| Carolina African American Twin Study of Aging | 73 | 176.7 | 8.3 | 28.4 | 5.5 |  | 65 | 174.8 | 8.8 | 28.3 | 5.5 |
| Colorado Twin Registry | 368 | 180.2 | 6.7 | 24.6 | 3.7 |  | 233 | 180.3 | 6.7 | 24.7 | 4.0 |
| Danish Twin Cohort | 6266 | 178.8 | 7.3 | 24.7 | 3.3 |  | 5410 | 179.4 | 7.1 | 24.8 | 3.4 |
| Adult Netherlands Twin Registry | 807 | 182.5 | 6.9 | 23.2 | 2.9 |  | 960 | 182.9 | 7.4 | 23.2 | 3.2 |
| Finnish Older Twin Cohort | 1152 | 174.3 | 6.3 | 26.2 | 3.2 |  | 1456 | 175.7 | 6.3 | 26.4 | 3.6 |
| FinnTwin12 | 456 | 179.3 | 6.7 | 24.5 | 3.5 |  | 480 | 178.0 | 6.6 | 24.4 | 3.4 |
| FinnTwin16 | 826 | 179.4 | 6.9 | 24.0 | 3.3 |  | 885 | 179.5 | 6.3 | 24.1 | 3.2 |
| East Flanders Prospective Twin Survey | 76 | 177.5 | 5.9 | 22.7 | 3.4 |  | 41 | 177.9 | 7.2 | 23.1 |  |
| Genesis 12-19 Study | 43 | 181.5 | 7.3 | 23.7 | 2.9 |  | 67 | 181.0 | 6.5 | 22.8 | 2.4 |
| Hungarian Twin Registry | 30 | 178.8 | 6.8 | 25.8 | 5.2 |  | 29 | 178.8 | 9.1 | 25.0 | 4.5 |
| Italian Twin Registry | 964 | 175.9 | 7.0 | 24.1 | 3.4 |  | 1074 | 176.2 | 7.6 | 24.0 | 3.5 |
| Mid-Atlantic Twin Registry | 1151 | 178.6 | 6.9 | 25.1 | 3.4 |  | 1334 | 178.3 | 7.1 | 25.1 | 3.6 |
| Minnesota Twin Registry | 1194 | 178.9 | 6.7 | 25.9 | 3.6 |  | 916 | 178.8 | 6.9 | 26.1 | 3.5 |
| Mongolian Twin Registry | 10 | 174.0 | 5.7 | 23.1 | 2.3 |  | 10 | 170.9 | 11.2 | 24.1 | 2.6 |
| Murcia Twin Registry | 322 | 171.1 | 7.9 | 27.7 | 4.1 |  | 270 | 170.3 | 7.2 | 28.0 | 4.4 |
| Qingdao Twin Registry (adults) | 92 | 169.2 | 6.9 | 24.1 | 3.0 |  | 94 | 169.8 | 6.7 | 24.3 | 3.3 |
| Queensland Twin Register | 2154 | 179.1 | 7.0 | 24.5 | 16.4 |  | 2180 | 178.9 | 6.8 | 24.3 | 3.4 |
| SRI-international | 44 | 178.9 | 5.1 | 25.4 | 3.5 |  | 42 | 179.2 | 6.9 | 25.8 | 4.0 |
| University of Southern California Twin Study | 33 | 174.9 | 8.7 | 23.8 | 4.4 |  | 16 | 176.6 | 9.2 | 25.6 | 5.9 |
| South Korea Twin Registry | 28 | 172.6 | 5.4 | 23.0 | 2.4 |  | 22 | 175.1 | 4.4 | 21.3 | 1.8 |
| Sri Lanka Twin Registry | 212 | 165.9 | 6.5 | 22.7 | 4.2 |  | 254 | 165.3 | 6.7 | 23.0 | 4.3 |
| Swedish Twin Cohorts | 6945 | 177.9 | 6.6 | 25.5 | 3.1 |  | 5581 | 177.9 | 6.5 | 25.6 | 3.1 |
| TCHAD-study | 51 | 179.5 | 5.4 | 22.5 | 2.4 |  | 42 | 179.6 | 5.7 | 23.3 | 1.7 |
| Turkish Twin Study | 92 | 174.0 | 7.6 | 24.3 | 3.2 |  | 50 | 172.7 | 6.8 | 23.8 | 3.0 |
| Washington State Twin Registry | 1183 | 179.6 | 7.6 | 26.21 | 4.2 |  | 1524 | 179.2 | 7.2 | 26.6 | 4.7 |
| West Japan Twins and Higher Order Multiple Births Registry | 13 | 172.6 | 5.6 | 21.2 | 2.8 |  | 16 | 170.0 | 7.3 | 21.1 | 2.4 |

BMI, body mass index; N, number of twin individuals; SD, standard deviation.

This descriptive table is not corrected for the non-independence (clustering) of observations within twin pairs.
