# Supplementary material for: Does the sex of one’s co-twin affect height and BMI in adulthood? A study of dizygotic adult twins from 31 cohorts
Source: Biol Sex Differ. 2017 Apr 27;8:14. doi: 10.1186/s13293-017-0134-x (PMC5408365; doi:10.1186/s13293-017-0134-x)
Supplement: Supplementary file 2 — The differences in height (cm) between opposite sex (OS) and same sex (SS) plotted against the mean heights (cm) of the cohorts for females and males. The figure examines whether the difference in height between OS and SS twins is greater in taller cohorts. The Spearman correlations are r = −0.16 and p = 0.39 for females and r = 0.10 and p = 0.59 for males. Figure S2. The differences in BMI (kg/m2) between opposite-sex (OS) and same-sex (SS) twins plotted against the mean BMIs (kg/m2) of the cohorts for females and males. The figure examines whether the difference in BMI between OS and SS twins is greater in heavier cohorts. The Spearman correlations are r = −0.39 and p = 0.029 in females and r = 0.10 and p = 0.61 for BMI in males. (DOCX 159 kb) [file 13293_2017_134_MOESM2_ESM.docx]

Additional file 2: Figure S1.
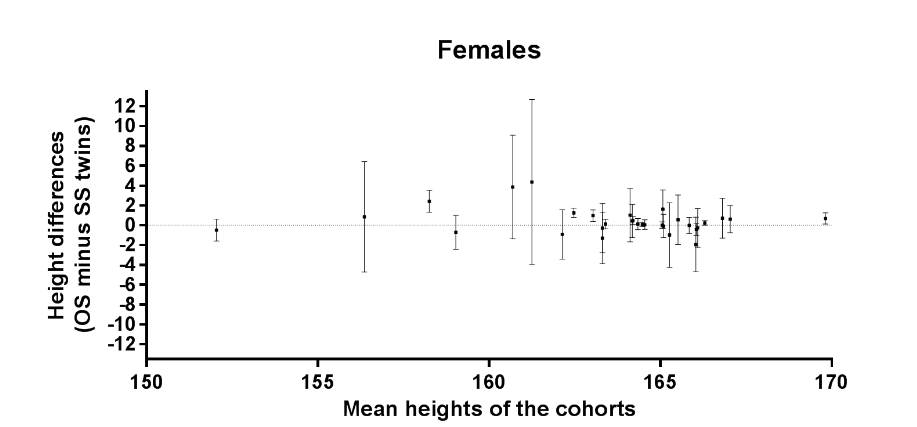


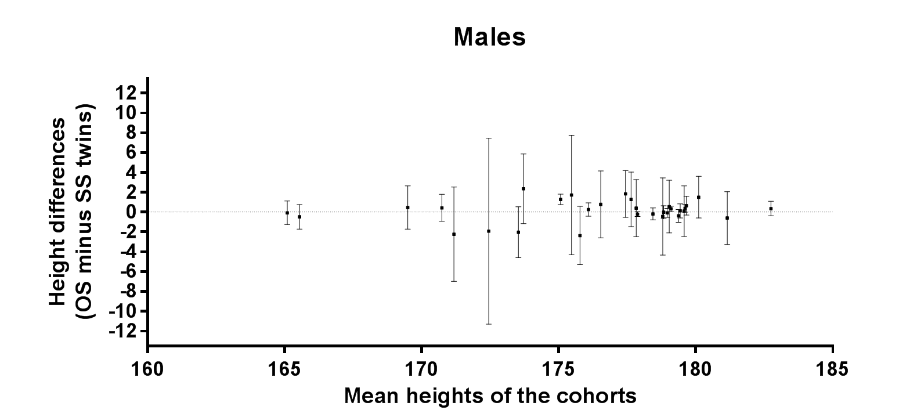


Figure legend. The differences in height (cm) between opposite sex (OS) and same sex (SS) plotted against the mean heights (cm) of the cohorts for females and males. The figure examines whether the difference in height between OS and SS twins is greater in taller cohorts. The Spearman correlations are r= -0.16, p=0.39 for females and r= 0.10, p=0.59 for males.

Additional file 2: Figure S2.


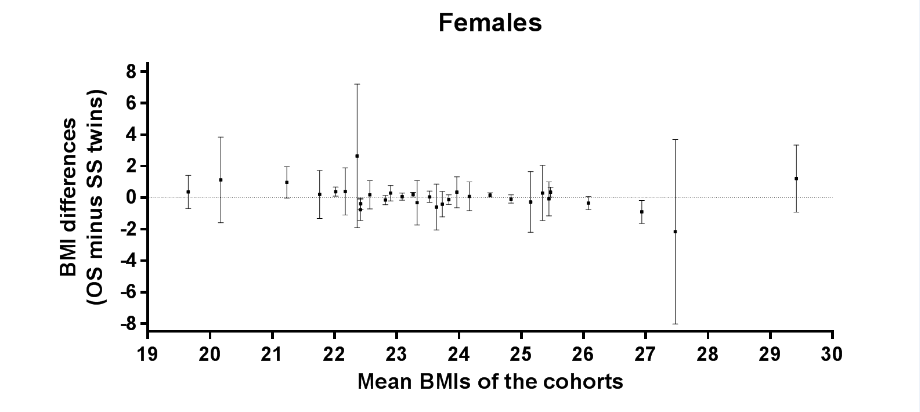


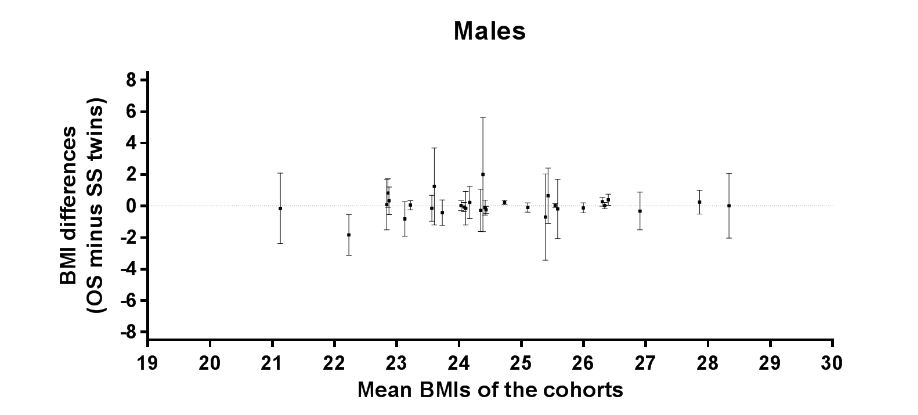


Figure legend. The differences in BMI (kg/m^2^) between opposite sex (OS) and same sex (SS) twins plotted against the mean BMIs (kg/m^2^) of the cohorts for females and males. The figure examines whether the difference in BMI between OS and SS twins is greater in heavier cohorts. The Spearman correlations are r= -0.39, p=0.029 in females, r= 0.10, p=0.61 for BMI in males.
